# KIF22 promotes the proliferation and glycolysis of melanoma by activating EGFR/STAT3 signaling

**DOI:** 10.1016/j.clinsp.2023.100307

**Published:** 2023-11-07

**Authors:** Zhi Zhong, Hua Zhong

**Affiliations:** a Department of Clinical Laboratory, Harbin Medical University Cancer Hospital, Harbin, Heilongjiang, China; b Department of Clean Operation, Harbin Medical University Cancer Hospital, Harbin, Heilongjiang, China

**Keywords:** KIF22, Melanoma, Proliferation, Glycolysis, EGFR/STAT3 signaling

## Abstract

**Objectives:**

Melanoma is one of the leading causes of cancer death. Kinesin Family member 22 (KIF22) is essential for the invasion of melanoma cells, but the role and mechanism of KIF22 in the proliferation and glycolysis in melanoma remains unknown.

**Methods:**

KIF22 expression in melanoma tissues and the relationship between KIF22 high expression and overall survival rate in patients with melanoma were analyzed using the Tnmplot database. KIF22 expression in melanoma cells was examined by western blot. Then, KIF22 was silenced and CCK-8 assay, EDU staining and flow cytometry analysis were adopted for assessing cell proliferation and apoptosis. In addition, the glycolysis metabolism of melanoma cells was reflected by detecting Extracellular Acidification Rates (ECAR) and Oxygen Consumption Rates (OCR). The expression of proteins related to apoptosis, glycolysis and EGFR/STAT3 signaling was tested by western blot. Subsequently, melanoma cells were treated with EGF or Colivelin to further elucidate the regulatory effect of KIF22 on EGFR/STAT3 signaling.

**Results:**

KIF22 expression was notably upregulated in melanoma tissues and cells, and KIF22 high expression was associated with a poor prognosis. Moreover, KIF22 insufficiency suppressed proliferation and accelerated apoptosis of melanoma cells. Additionally, glycolysis was reduced by KIF22 depletion, evidenced by the decreased ECAR and increased OCR, accompanied by the downregulated expression of HK2, PKM2 and LDHA. Importantly, the impacts of KIF22 depletion on the progression of melanoma were partially attenuated after EGF or Colivelin treatment.

**Conclusion:**

Collectively, KIF22 knockdown suppressed the proliferation and glycolysis and facilitated the apoptosis of melanoma cells by inactivating EGFR/STAT3 signaling.

## Highlights

• KIF22 expression is upregulated in melanoma tissues and cells.
• KIF22 knockdown suppresses proliferation and facilitates apoptosis of melanoma cells.
• KIF22 knockdown inhibits tumor glycolysis in melanoma cells.
• KIF22 depletion suppresses proliferation, glycolysis and facilitates apoptosis of melanoma cells by inactivating EGFR/STAT3 signaling.

## Introduction

Malignant melanoma, an aggressive cancer originating from cells that produce melanin, is the leading cause of death in patients with skin tumors.^1^ As reported, the morbidity of melanoma has been continuously increasing every year worldwide.^2^ Despite the great improvements in medical standards and treatment options available to melanoma patients, the mortality rate for melanoma remains high, especially for patients with metastasis.^3^^,^^4^ The prognosis of melanoma remains dismal due to its proclivity to metastasize. Therefore, it is extremely urgent for us to deepen our understanding of the molecular mechanisms related to the progression of melanoma and to develop more promising therapeutic targets for melanoma.

Increasing evidence suggests that interfering with tumor energy metabolism has become an effective means of controlling cancer progression.^5^^,^^6^ As the main form of metabolic reprogramming in cancer, aerobic glycolysis, also known as the Warburg effect, supplies energy for tumor rapid growth by prioritizing the intake of glucose as the primary energy source.^7^ Aerobic glycolysis is one of the most important hallmarks of cancers including melanoma and the inhibition of the glycolysis process has been recognized as an effective strategy to treat melanoma.^8^^,^^9^ Kinesin Family member 22 (KIF22), a Kinesin-like DNA binding protein (KID) belonging to the kinesin-10 family, is considered significant in tumorigenesis and cancer progression.^10^ A growing number of studies have evidenced that KIF22 has significantly high expression in several tumor types, such as esophageal squamous cell carcinoma, gastric cancer, and bladder cancer.^11^, ^12^, ^13^ It is worth noting that KIF22 is required for the invasion of melanoma cells, and the knockdown of KIF22 inhibits the invasion of melanoma cells.^14^ So far, the effects of KIF22 on the proliferation and glycolysis of melanoma cells have not been investigated. It has been reported that KIF22 can activate Epidermal Growth Factor Receptor (EGFR) signaling, an effective target for melanoma.^15^^,^^16^ Additionally, EGFR-mediated Signal Transduction and Transcription Factor 3 (STAT3) signaling activation promote cell proliferation and glycolysis in various cancers.^17^^,^^18^ Therefore, the authors hypothesized that KIF22 might affect the proliferation and glycolysis of melanoma cells by regulating EGFR/STAT3 signaling.

In this study, KIF22 expression in melanoma tissues and the correlation between KIF22 expression and prognosis were assessed by bioinformatics tools. The effects of KIF22 insufficiency on the proliferation and glycolysis in melanoma cells were analyzed. Further, the potential mechanisms of KIF22 related to EGFR/STAT3 signaling were explored in the subsequent experiments. The present findings might provide a novel molecular mechanism for further understanding the progression of melanoma.

## Materials and methods

### Bioinformatics databases

KIF22 expression in melanoma tissues (Tumor) and normal tissues as well as the relationship between KIF22 high expression and overall survival rate in patients with melanoma was analyzed by using the Tnmplot database (https://tnmplot.com/analysis/).

### Cell culture

Human malignant melanoma cell lines SK-MEL-1, SK-MEL-28 and A375 were obtained from BeNa Culture Collection (Beijing, China). MeWo cells were acquired from the American Type Culture Collection (Manassas, VA, USA). The normal human epidermal melanocytes (HEM) were bought from ScienCell (Carlsbad, CA, USA). These cells were grown in Dulbecco's modified Eagle's medium (DMEM; Gibco, Carlsbad, CA, USA) supplemented with 10% fetal bovine serum (FBS; Gibco, Carlsbad, CA, USA) at 37°C with 5% CO_2_. To investigate the mechanism of KIF22 associated with EGFR/STAT3 signaling in melanoma, A375 cells were treated with 25 ng/mL EGF (Sigma-Aldrich) or 0.5 μM Colivelin (MedChem Express, Princeton, NJ).

### Cell transfection

Transfection was performed by using Lipofectamine 3000 (Invitrogen, USA) when cell confluence reached 80%. The small interfering RNAs against KIF22 (siRNA-KIF22-1, 5’-GGGAAAACTCTACCTGATTGACT-3; siRNA-KIF22-1, 5’-TAGAGATTGAGAGGCTTAAGACG-3) and negative control (siRNA-NC) were supplied by GenePharma (Shanghai, China). The transfected cells were cultivated for 48h and the successful transfection was evaluated by using Real‑Time quantitative PCR (RT-qPCR) and western blot.

### RT-qPCR

The total RNA was extracted from A375 cells using Trizol reagent (Takara, Toyobo, Japan). The complementary DNA (cDNA) was synthesized using the PrimeScript RT reagent kit (Takara, Toyobo, Japan). qPCR was performed on the ABI 7500 Real-Time PCR system (Applied Biosystems, Foster City, CA) with cDNA as templates using SYBR Green PCR Master Mix Reagents (Takara, Toyobo, Japan) based on the recommendations provided by the manufacturer. The primer sequences were listed as follows: KIF22 forward, 5’-TTCTTGAGGGCCTACTCTGC-3’, and reverse, 5’-GGTCCATAGGCAAGCACACT-3’; β-actin forward, 5’-GCCTCGCCTTTGCCGAT-3’, and reverse 5’-AGGTAGTCAGTCAGGTCCCG-3’. The relative gene expression was determined with 2^−△△CT^ method (Livak and Schmittgen 2001). β-actin was employed as the internal reference.

### Immunoblotting

Total protein in cells was extracted using RIPA buffer (Beyotime, Shanghai, China) and the protein concentration was quantified with the application of a BCA Kit (Beyotime, Shanghai, China) as per the manufacturer's instructions. Equal amounts of proteins were separated by electrophoresis on 10% SDS-PAGE gels and then transferred onto PVDF membranes. After the blockade with 5% BSA for 2h, the membranes were probed with primary antibodies overnight at 4°C, followed by the incubation with HRP-conjugated secondary antibody at room temperature for 1h. The protein bands were examined by chemiluminescence reagents (Millipore, Burlington, MA, USA) and imaged by film development. The intensities of the bands were detected by ImageJ Software. The relative protein expression was calculated with β-actin being the loading control.

### Cell viability assay

Cell viability was assessed using the Cell Counting Kit-8 (CCK-8) assay (Beyotime; Shanghai, China). After the indicated treatment, CCK-8 solution was added to each well to incubate cells for another 4h at 37°C. The optical density was detected using a microplate reader (Biotek, USA) at a wavelength of 450 nm.

### 5-Ethynyl-2’-deoxyuridine (EDU) staining

Cell proliferation was evaluated using an EDU kit (Beyotime Biotech, Haimen, China) according to the manufacturer's protocol. A375 cells were injected into 12-well plates and then cultured until cell confluence reached 70%. After the indicated treatment, cells were incubated with EDU for 2h before the fixation with 4% paraformaldehyde and the permeation with 0.3% Triton X-100. Cell nuclei were labeled with 4, 6-Diamino-2-Phenylindole (DAPI) in the dark. Finally, the proliferation of A375 cells was observed and imaged by an inverted fluorescence microscope (Olympus, Tokyo, Japan).

### Apoptosis analysis

The apoptosis of A375 cells with indicated treatment was assessed by using annexin V Fluorescein Isothiocyanate (FITC)/Propidium Iodide (PI) labeling (Vazyme). A375 cells were washed twice with ice-cold PBS, and then stained with 5 μL of annexin V-FITC and 5 μL of PI for 15 min away from light. The staining results were analyzed by a flow cytometer (BD Biosciences, San Jose, CA, USA)

### Glycolysis analysis

By means of the Seahorse XF glycolytic Stress Test kit and the Seahorse XF Cell Mito Stress Test Kit in the Seahorse XFe96 analyzer (Seahorse Bioscience, USA), extracellular acidification rate (ECAR, mpH/min) and oxygen consumption rates (OCR, pmoL/min) were calculated as per the manufacturer's instructions. Seahorse XF-96 Wave software was used for data analysis.

### Measurement of lactate secretion

The level of lactate secretion in the medium was detected using a lactate assay kit (Nanjing Jiancheng Bioengineering Institute, Nanjing, China) according to the manufacturer's protocol. Briefly, 1 mL of enzyme working solution and 0.2 mL of chromogenic reagent were added to 0.02 mL sample. After mixing, the samples were reacted at 37°C for 10 min. Then, 2 mL of termination solution was added and mixed. The absorbance value at 530 nm was tested using a microplate reader (Biotek, USA).

### Statistical analysis

Statistical analysis was performed using GraphPad 8.0 software (GraphPad Software Inc., USA) and then presented as mean ± Standard Deviation (SD). One-way analysis of variance followed by Tukey's post hoc test and Student's *t*-test were used for comparison procedure. Significance is determined when the p-value is less than 0.05.

## Results

### KIF22 expression is upregulated in melanoma tissues and cells

**Fig. 1:**
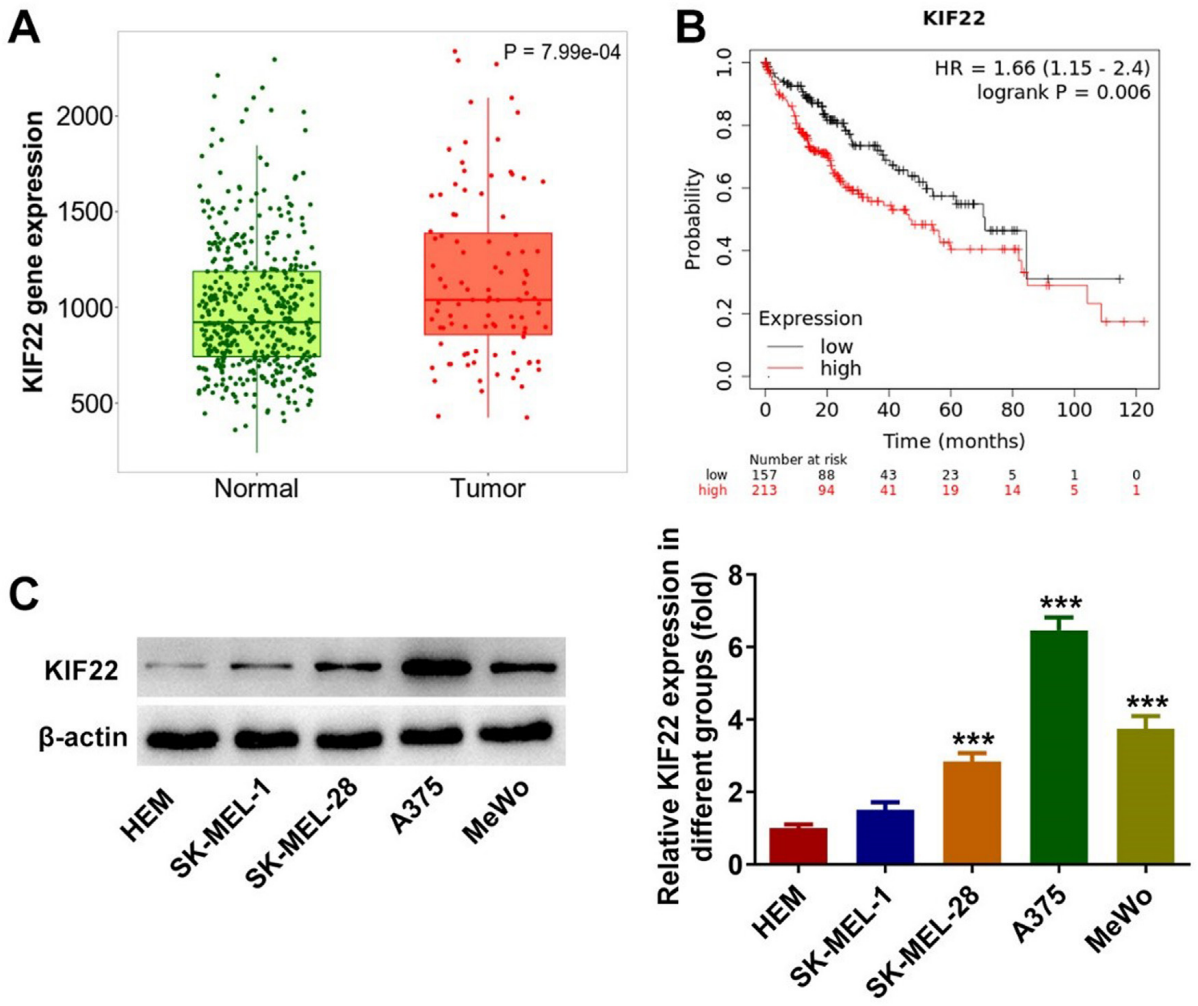
KIF22 expression was upregulated in melanoma tissues and cells. (A) KIF22 expression in melanoma tissues (Tumor) and normal tissues was analyzed by using Tnmplot database. (B) The relationship between KIF22 high expression and overall survival rate in patients with melanoma was evaluated by using the Tnmplot database. (C) KIF22 expression in various melanoma cell lines (SK-MEL-1, SK-MEL-28, A375 and MeWo) and the normal Human Epidermal Melanocytes (HEM) was detected using western blot. *** p < 0.001 vs. HEM.

To figure out the role of KIF22 in melanoma, the Tnmplot database was initially employed to evaluate KIF22 expression in melanoma tissues and analyze the relationship between KIF22 high expression and the overall survival rate in melanoma patients. It was found that KIF22 was highly expressed in melanoma tissues compared with the normal group, and higher KIF22 expression was associated with a lower overall survival rate (Fig. 1 A‒B). Additionally, a western blot was used to assess KIF22 expression in various melanoma cell lines (SK-MEL-1, SK-MEL-28, A375 and MeWo) and the normal Human Epidermal Melanocytes (HEM). As exhibited in Fig. 1C, KIF22 expression in SK-MEL-28, A375 and MeWo cells was significantly elevated compared with the HEM group. Compared with SK-MEL-28 and MeWo groups, KIF22 had the highest expression level in A375 cells, in view of this, A375 cells were chosen for the following experiments. These data suggest that KIF22 may play a promoting role in melanoma.

### KIF22 knockdown suppresses proliferation and facilitates apoptosis of melanoma cells

**Fig. 2:**
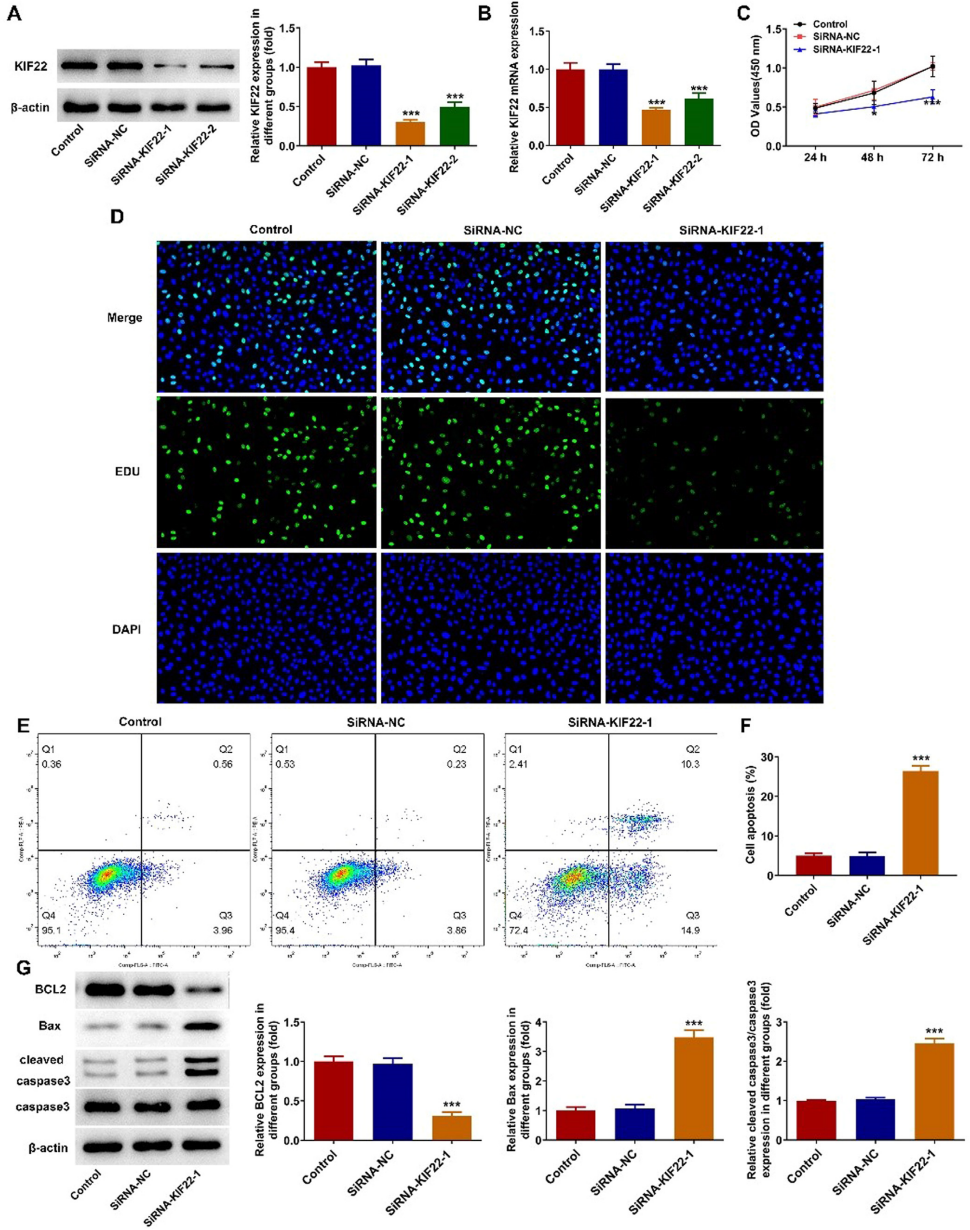
KIF22 knockdown suppressed proliferation and facilitated apoptosis of melanoma cells. (A) KIF22 protein expression in A375 cells after transfection was estimated with the help of western blot. (B) KIF22 mRNA expression in A375 cells after transfection was tested by RT-qPCR. (C) CCK-8 assay was utilized to assess the viability of KIF22-silenced A375 cells. (D) EDU staining was used to evaluate the ability of A375 cell proliferation after transfection. (E) The apoptosis of KIF22-silenced A375 cells was tested by using flow cytometry assay. (F) Quantification of apoptosis was detected by flow cytometry assay. (G) The expression of apoptosis-related proteins in KIF22-silenced A375 cells was detected by western blot. * p < 0.05, *** p < 0.001 vs. siRNA-NC group.

To explore the role of KIF22 in the malignant phenotypes of melanoma cells, siRNA-KIF22-1/2 was transfected into A375 cells to silence KIF22 expression. Compared with the siRNA-NC group, KIF22 mRNA and protein expression was notably reduced in A375 cells after the transfection with siRNA-KIF22-1/2 (Fig. 2 A‒B). SiRNA-KIF22-1 was selected for subsequent experiments due to its better silencing efficiency. CCK-8 assay proved that interference with KIF22 decreased the viability of A375 cells (Fig. 2C). Consistently, the EDU fluorescence intensity in A375 cells was prominently diminished in the siRNA-KIF22-1 group relative to the siRNA-NC group, suggesting the inhibitory effect of KIF22 knockdown on the proliferation of melanoma cells (Fig. 2D). Besides, cell apoptosis was detected using flow cytometry assay. The apoptotic rate was elevated after silencing KIF22 in A375 cells, accompanied by the decreased expression level of BCL2 and increased expression levels of Bax and cleaved caspase 3 (Fig. 2 E‒G). These results demonstrate that KIF22 insufficiency inhibits the proliferation and facilitates the apoptosis of melanoma cells.

### KIF22 knockdown inhibits tumor glycolysis in melanoma cells

**Fig. 3:**
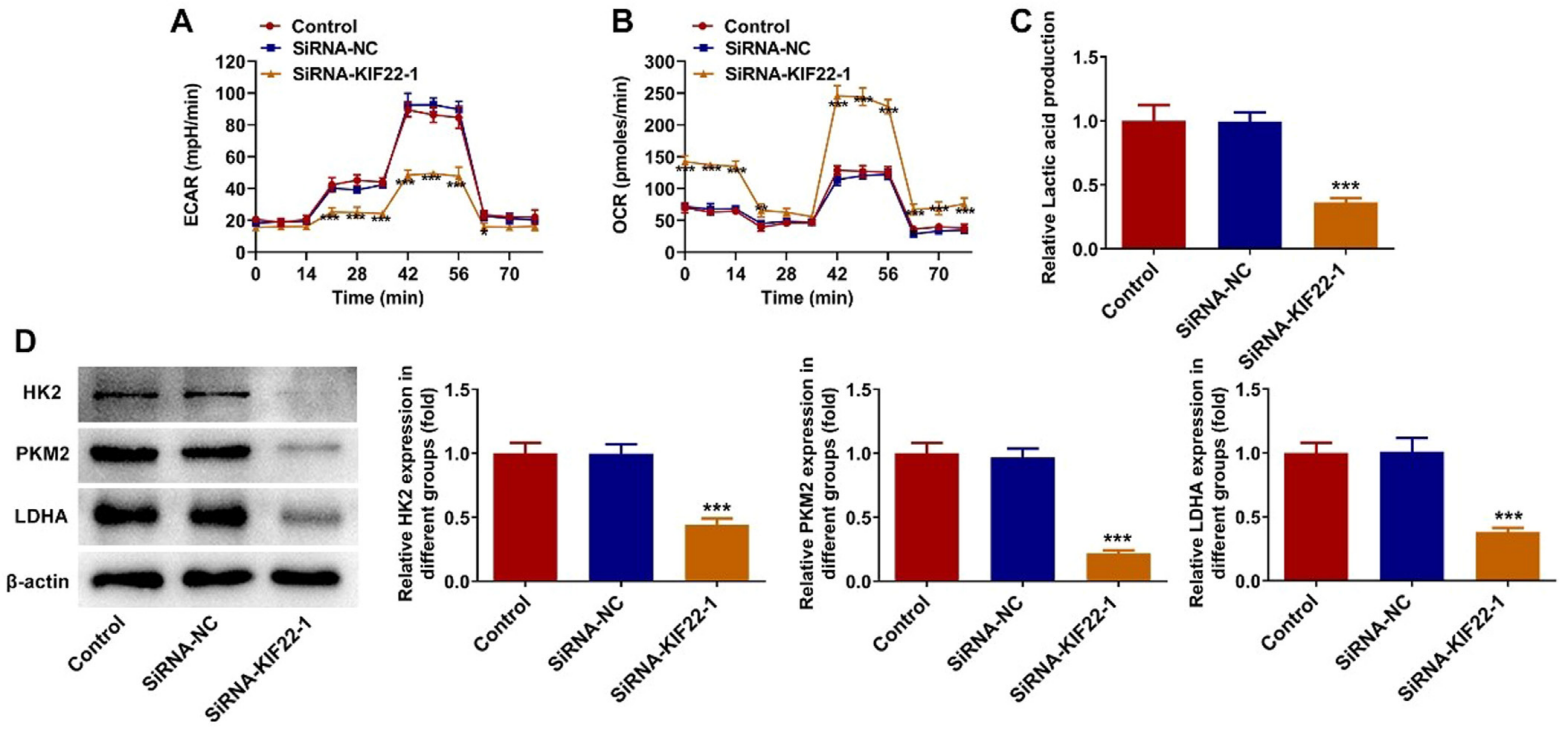
KIF22 knockdown inhibited tumor glycolysis in melanoma cells. (A) The level of ECAR in KIF22-silenced A375 cells was assessed by the commercially available kit. (B) The level of OCR in KIF22-silenced A375 cells was assessed by the commercially available kit. (C) Lactate production in A375 cells after transfection was tested by the commercially available kit. (D) The expression of HK2, PKM2 and LDHA was examined by western blot. *p < 0.05, ^⁎⁎^p < 0.01, ^⁎⁎⁎^p < 0.001 vs. siRNA-NC group.

To explore whether KIF22 depletion could decrease aerobic glycolysis in melanoma, the changes in the levels of glycolysis-related metabolites were detected in this section. Firstly, the levels of ECAR, which reflects the aerobic glycolytic flux, and OCR, which indicates the state of mitochondrial oxidative respiration, were measured by using commercially available kits. As presented in Fig. 3 A‒B, compared with the siRNA-NC group, KIF22 knockdown led to a remarkable decrease in ECAR level and a significant increase in OCR level. Moreover, the lactate secretion level was also significantly reduced in KIF22-silenced A375 cells (Fig. 3C). Besides, KIF22 depletion markedly downregulated the expression of Hexokinase (HK) 2, Pyruvate Kinase Muscle isozyme M2 (PKM2) and Lactate Dehydrogenase A (LDHA), which were key enzymes involved in the progression of glycolysis (Fig. 3D). Collectively, these results imply the inhibitory role of KIF22 knockdown on tumor glycolysis in melanoma cells.

### KIF22 depletion suppresses proliferation, glycolysis and facilitates apoptosis of melanoma cells by inactivating EGFR/STAT3 signaling

**Fig. 4:**
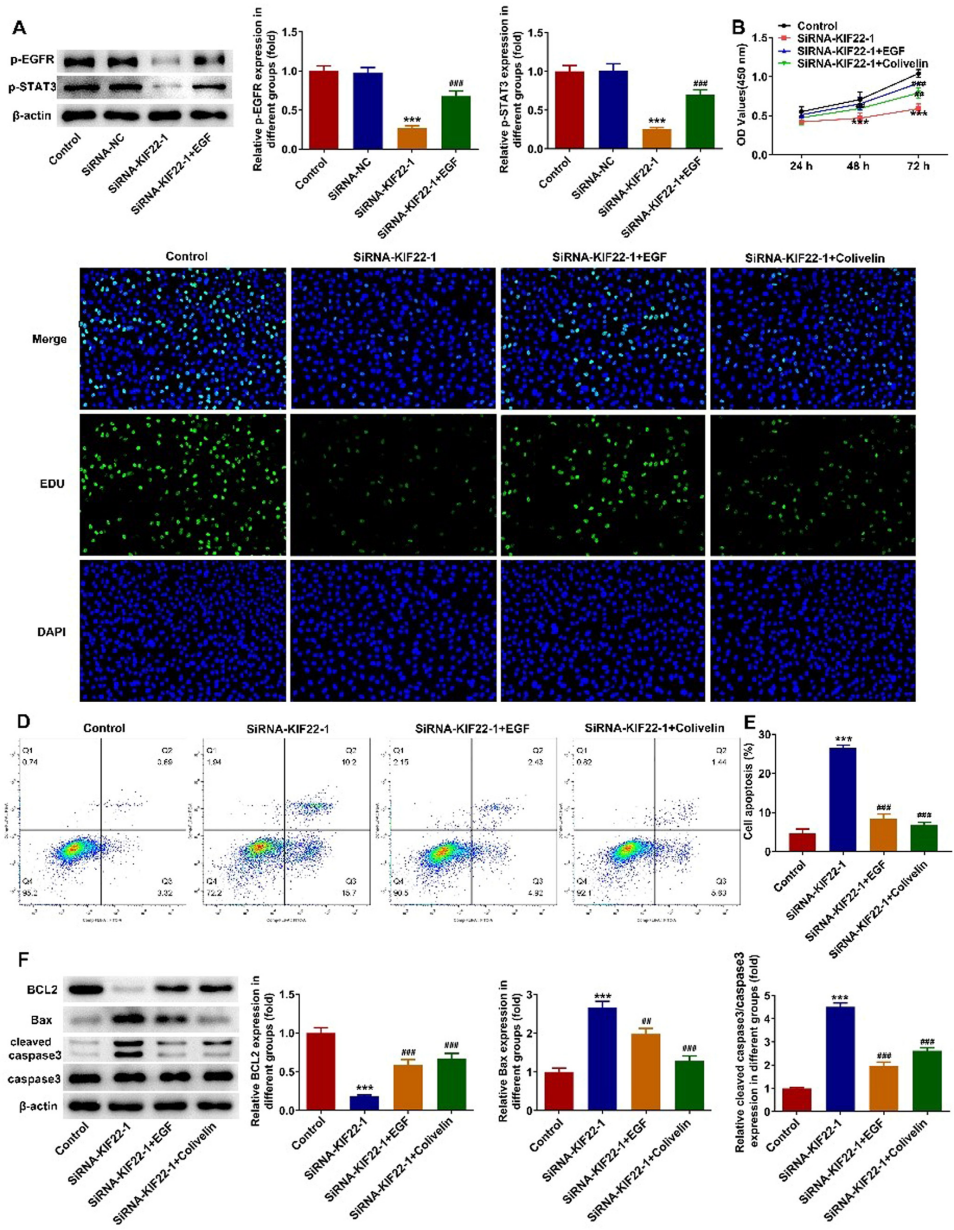
KIF22 knockdown suppressed proliferation and aggravated apoptosis of melanoma cells by inactivating EGFR/STAT3 signaling. (A) The expression of p-EGFR and p-STAT3 in KIF22-silenced A375 cells with or without EGF treatment was detected by western blot. ^⁎⁎⁎^ p < 0.001 vs. siRNA-NC; ^###^ p < 0.001 vs. siRNA-KIF22. (B) CCK-8 assay was utilized to assess the viability of KIF22-silenced A375 cells with or without EGF/Colivelin treatment. (C) EDU staining was used to evaluate the proliferation of KIF22-silenced A375 cells with or without EGF/Colivelin treatment. (D) The apoptosis of KIF22-silenced A375 cells with or without EGF/Colivelin treatment was tested by using flow cytometry assay. (E) Quantification of apoptosis was detected by flow cytometry assay. (F) The expression of apoptosis-related proteins in KIF22-silenced A375 cells with or without EGF/Colivelin treatment was detected by western blot. ^⁎⁎⁎^ p < 0.001 vs. control; ^##^ p < 0.01, ^###^ p < 0.001 vs. siRNA-KIF22.

**Fig. 5:**
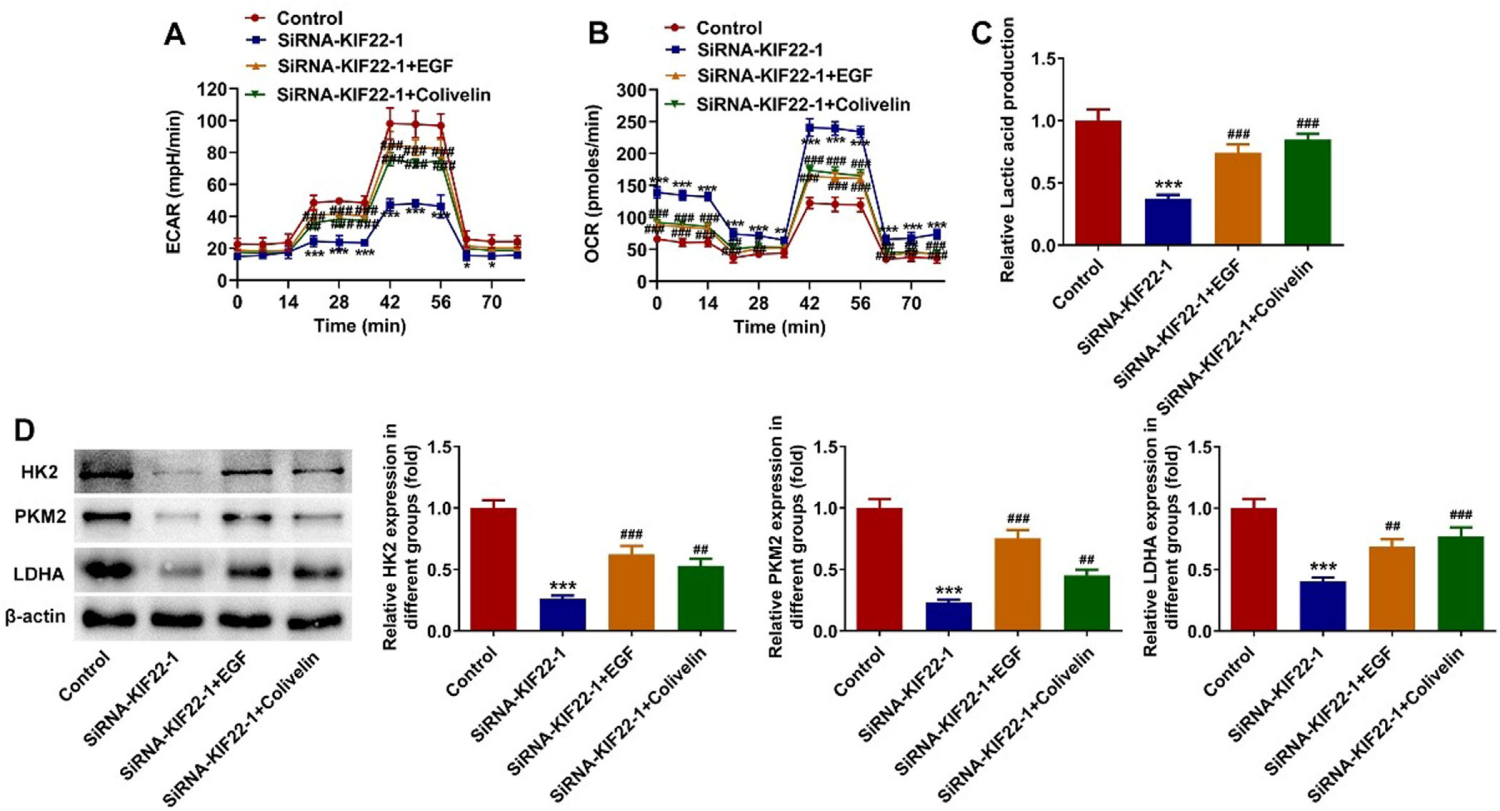
KIF22 knockdown facilitated tumor glycolysis in melanoma cells by inactivating EGFR/STAT3 signaling. (A) The level of ECAR in KIF22-silenced A375 cells with or without EGF/Colivelin treatment was assessed by the commercially available kit. (B) The level of OCR in KIF22-silenced A375 cells with or without EGF/Colivelin treatment was assessed by the commercially available kit. (C) Lactate production in KIF22-silenced A375 cells with or without EGF/Colivelin treatment was tested by the commercially available kit. (D) The expression of HK2, PKM2 and LDHA in KIF22-silenced A375 cells with or without EGF/Colivelin treatment was examined by western blot. ^⁎⁎^ p < 0.01, ^⁎⁎⁎^ p < 0.001 vs. control; ^#^ p < 0.05, ^##^ p < 0.01, ^###^ p < 0.001 vs. siRNA-KIF22.

To elucidate the cellular mechanism underlying the inhibitory effect of KIF22 depletion on the malignant phenotypes of melanoma cells, the role of KIF22 in EGFR/STAT3 signaling was investigated and western blot was adopted for the evaluation of proteins involved in this pathway with or without EGF treatment. It was observed that KIF22 deficiency significantly downregulated the expression of p-EGFR and p-STAT3 by contrast with the siRNA-NC group, which was partly restored by EGF addition (Fig. 4A). Subsequently, A375 cells were treated with 0.5 μM Colivelin, an activator of STAT3. As exhibited in Fig. 4 B‒C, EGF or Colivelin treatment abolished the inhibitory effects of KIF22 depletion on the viability and proliferation of A375 cells. In addition, the elevated apoptotic rate of A375 cells induced by KIF22 knockdown was reduced by EGF or Colivelin (Fig. 4 D‒E). Consistently, the addition of EGF or Colivelin remarkably upregulated BCL2 expression and downregulated Bax and cleaved caspase3 expression compared with the siRNA-KIF22 group (Fig. 4F). Besides, the impacts of KIF22 depletion on the levels of ECAR, OCR, lactate secretion, HK2, PKM2 and LDHA expression were partially counteracted after the treatment with EGF or Colivelin (Fig. 5 A‒D). Summing up, KIF22 depletion represses proliferation, inhibits glycolysis, and accelerates apoptosis of melanoma cells by inactivating EGFR/STAT3 signaling.

## Discussion

Melanoma is the most devastating form of skin cancer, contributing to the largest number of skin cancer-related deaths globally. The process of tumorigenesis involves a variety of oncogenes and tumor suppressor genes, and the changes in these genes lead to the formation of tumors. At present, melanoma has been extensively studied, but its underlying mechanism is not well understood. The present study demonstrated that KIF22 was upregulated in melanoma tissues and cells. The authors revealed for the first time that KIF22 depletion could restrain proliferation, glycolysis and accelerate apoptosis of melanoma cells by inactivating EGFR/STAT3 signaling.

The uncontrolled rapid cell proliferation has been recognized as an important feature of tumor cells and pathways that limit the proliferative response of normal cells are disrupted during tumor development. Apoptosis exerts crucial effects in the development of multicellular organisms, which directly affects the regulation and maintenance of cell populations.^19^ Therefore, targeting cell proliferation and apoptosis has become an attractive intervention strategy for cancer therapy. KIF22 is a member of the kinesin family and is involved in spindle formation during mitosis.^20^ As an oncogene, KIF22 has been demonstrated to be highly expressed in multiple human cancers. For instance, Yu et al. have found that the suppression of KIF22 reduces cell proliferation in cancer by delaying mitotic exit.^21^ Liu and colleagues have reported that KIF22 is significantly elevated in the tissues of tongue squamous cell carcinoma, and KIF22 inhibition notably restrains the proliferation of cancer cells both in vitro and in vivo.^22^ In addition, the abnormally overexpressed KIF22 in gastric cancer tissues is remarkably related to the poor prognosis of gastric cancer patients and silencing KIF22 can reduce the proliferation of gastric cancer cells.^12^ Particularly, KIF22 is required for the invasion of melanoma cells, and the knockdown of KIF22 inhibits the invasion of melanoma cells.^14^ In this study, the authors discovered that KIF22 was overexpressed in melanoma tissue and high KIF22 expression was positively associated with lower overall survival according to the Tnmplot database. Of note, the authors also demonstrated for the first time that KIF22 insufficiency suppressed the proliferation of melanoma cells.

Cancer cells tend to rely on glycolysis rather than oxidative phosphorylation to generate energy, and this metabolic reprogramming is the major metabolic mode by which most tumor cells generate energy for proliferation.^23^ In the process of glycolytic metabolism, cancer cells use glucose as the main energy source, and lactic acid is the main product.^24^ Aerobic glycolysis is one of the most important hallmarks of cancer including melanoma and the inhibition of the glycolysis process has been considered to be the basis of tumor cell survival and tumorigenesis.^8^^,^^25^ ECAR and OCR are two key markers for assessing the levels of aerobic glycolysis and oxidative respiration, respectively.^26^ In addition, the process of glycolysis is catalyzed by some rate-limiting enzymes. HK2 is a key enzyme that regulates the first step of glycolysis.^27^ PKM2, as the most critical rate-limiting enzyme,^29^ mediates the last step of the glycolysis.^28^ Besides, LDHA plays a crucial role in glycolysis, which is the final step in aerobic lactate production.^29^ In this work, KIF22 interference notably repressed glycolysis in melanoma cells, evidenced by reduced levels of ECAR, lactate production, HK2, PKM2 and LDHA expression as well as elevated levels of OCR.

Increasing evidence indicates that EGFR signaling is important for the regulation of multiple cellular processes and is abnormally activated in many tumors.^30^ Therefore, targeted therapy targeting the EGFR signaling has become the focus of drug development for cancer treatment.^31^ EGFR-mediated STAT3 signaling activation promotes the proliferation and glycolysis in various cancers.^17^^,^^18^ In the study of melanoma, several reports have demonstrated that the activation of EGFR/STAT3 signaling promotes the malignant progression of melanoma.^32^^,^^33^ It has been reported that KIF22 activates EGFR signaling, an effective target for melanoma.^15^^,^^16^ Based on these results, the authors demonstrated that KIF22 knockdown inhibited the EGFR/STAT3 signaling in A375 cells. Importantly, the authors also found that the impacts of KIF22 depletion on the proliferation, apoptosis and glycolysis in A375 cells were partially attenuated after the treatment with EGF or Colivelin, suggesting that KIF22 silencing impeded the progression of melanoma by activating EGFR/STAT3 signaling.

## Conclusion

Taken together, the present study is the first to demonstrate that KIF22 knockdown suppresses the proliferation and glycolysis and facilitates the apoptosis of melanoma cells by inactivating EGFR/STAT3 signaling. The present study sheds novel insights into the understanding of melanoma progression and provides potential therapeutic targets for the targeted therapies of melanoma.

## Authors’ contributions

ZZ and HZ designed the study, and acquired, analyzed, and interpreted the data. HZ wrote the manuscript and ZZ revised it. All of the authors confirmed the authenticity of all the raw data and read and approved the final manuscript.

## Funding

Not applicable.

## Availability of data and materials

The experimental data are available from the corresponding author on reasonable request.

## Conflicts of interest

The authors declare no conflicts of interest.
